# Confirmation of brain death using optical methods based on tracking of an optical contrast agent: assessment of diagnostic feasibility

**DOI:** 10.1038/s41598-018-25351-6

**Published:** 2018-05-09

**Authors:** Wojciech Weigl, Daniel Milej, Anna Gerega, Beata Toczyłowska, Piotr Sawosz, Michał Kacprzak, Dariusz Janusek, Stanisław Wojtkiewicz, Roman Maniewski, Adam Liebert

**Affiliations:** 1 Anesthesiology and Intensive Care, Department of Surgical Sciences, Uppsala University, Akademiska Hospital, 751 85 Uppsala, Sweden; 2 Medical University of Warsaw, First Department of Anesthesiology and Intensive Care, Lindleya 4 st., Warsaw, Poland; 3 Nalecz Institute of Biocybernetics and Biomedical Engineering, Polish Academy of Sciences, Ks. Trojdena 4 st., 02-109 Warsaw, Poland

## Abstract

We aimed to determine whether optical methods based on bolus tracking of an optical contrast agent are useful for the confirmation of cerebral circulation cessation in patients being evaluated for brain death. Different stages of cerebral perfusion disturbance were compared in three groups of subjects: controls, patients with posttraumatic cerebral edema, and patients with brain death. We used a time-resolved near-infrared spectroscopy setup and indocyanine green (ICG) as an intravascular flow tracer. Orthogonal partial least squares-discriminant analysis (OPLS-DA) was carried out to build statistical models allowing for group separation. Thirty of 37 subjects (81.1%) were classified correctly (8 of 9 control subjects, 88.9%; 13 of 15 patients with edema, 86.7%; and 9 of 13 patients with brain death, 69.2%; p < 0.0001). Depending on the combination of variables used in the OPLS-DA model, sensitivity, specificity, and accuracy were 66.7–92.9%, 81.8–92.9%, and 77.3–89.3%, respectively. The method was feasible and promising in the demanding intensive care unit environment. However, its accuracy did not reach the level required for brain death confirmation. The potential usefulness of the method may be improved by increasing the depth of light penetration, confirming its accuracy against other methods evaluating cerebral flow cessation, and developing absolute parameters for cerebral perfusion.

## Introduction

The diagnosis of brain death is primarily clinical and is based on highly specific neurological examination of the patient. However, clinical determination of death can be hindered by numerous confounders, such as intoxication, severe craniofacial trauma, high cervical spine injuries^1,2^, or possible mimickers of life, i.e., complex motor movements^3^. In situations wherein accurate clinical evaluation is doubtful, ancillary tests are used to confirm diagnosis^4^. These tests are even mandatory in some countries irrespective of the results of the neurological examination^5,6^. Declaration of death is a challenging practice^7,8^, and recent controversial case reports^9–11^ have driven some clinicians to advocate for mandatory ancillary tests for the diagnosis of death even when there is no such statutory requirement^7,12^.

Ancillary tests used to prove blood flow cessation, such as four-vessel angiography or radionuclide imaging, are currently the most recommended methods^13–15^. However, there are numerous limitations to these tests. These include the need to transport the patient outside the intensive care unit (ICU) for completion of the investigation, availability of highly competent medical staff, and in the case of transcranial Doppler, unclear sensitivity^16^. An ancillary test should ideally be fast and performed at the bedside, readily available, non-invasive, and inexpensive^17^.

Optical methods based on illumination of the human head by light may meet the above-mentioned criteria. Due to limited absorption of light at near-infrared wavelengths and pronounced scattering, photons can penetrate through the skull and cerebral tissue. They can then be detected in reflectance geometry a few centimeters apart from the emission point on the surface of the head. Oxyhemoglobin and deoxyhemoglobin are the main chromophores contained in cerebral tissue that strongly absorb light in the near-infrared wavelength range. Changes in the concentrations of these chromophores are indicative of the oxygenation level of the blood passing through the brain, and can be monitored using near-infrared spectroscopy (NIRS). Until now, a few NIRS studies assessing cerebral oximetry as an indicator of cerebral blood flow (CBF) have been performed for confirmation of brain death^18,19^. Regional cerebral tissue oxygen saturation (rSO_2_), which is typically estimated in NIRS studies, depends on oxygen saturation of arterial and venous blood, as well as the composition of the volumes of the arterial, venous, and capillary vascular compartments. The relationship between rSO_2_ and CBF is complex, especially when cerebral metabolism is no longer maintained. There are additional factors influencing rSO_2_ in patients with brain death when residual CBF is preserved or when steal phenomena of blood from extracerebral tissues to cortex capillaries is present^20^. Furthermore, NIRS has the disadvantage of the unreliable separation of information from intracerebral tissue and extracerebral contamination^21^. Consequently, measurements of rSO_2_ have not proven useful for the confirmation of brain death, as the values of cerebral oximetry in some cases remain within the normal range even when cessation of CBF is confirmed^18,19,22^.

A potential new method for the determination of CBF appeared with the application of optical methods and bolus-tracking techniques using indocyanine green (ICG) as an intravascular flow tracer. It was shown that ICG concentration changes can be monitored during its circulation through the brain, and several algorithms have been used to assess CBF^23–25^. It was reported that time-resolved NIRS (TR-NIRS) can be utilized to track ICG with improved depth discrimination^26,27^. TR-NIRS is based on the emission of short laser light pulses into the tissue and the analysis of the distributions of times of flight of photons reemitted from the tissue. The method allows for selective analysis of the late photons, which have a higher probability of penetrating the cerebral cortex. This technique thus allows for more selective brain probing as confirmed in several methodological experiments using TR-NIRS^28,29^, in measurements carried out on animals^27^, in patients with stroke^30,31^ and during carotid surgery^32^. Subsequently, a time-resolved method allowing for the detection of fluorescence photons excited in the ICG during circulation of the dye through the tissues was proposed. This method was validated in a series of experiments on healthy subjects^33^ and was applied together with the diffuse reflectance method in patients with traumatic brain injury^34^. In the present study, we investigated whether these optical methods are useful for the confirmation of cessation of cerebral circulation in patients who undergo determination of brain death.

## Results

Sixteen patients were included in the study group. In 13 of these patients, measurements were performed during the study period. One patient was excluded due to liver insufficiency, one due to hemodynamic instability, and one due to personal unavailability. Mechanisms of cerebral injury and patient characteristics are presented in Table 1.

**Table 1:** Baseline demographic and clinical characteristics of study groups.

No.		Age/years	Sex		Age/years	Sex	GCS	MLS (mm)	Discharge status		Age/years	Sex	Mechanism of cerebral injury	Day in the ICU	Brain death confirmation method
1	Healthy volunteers	29	M	Patients with posttraumatic cerebral edema	48	M	3	2	Died	Patients with brain death	50	F	TBI	2	CE + CT perfusion
2		46	M		26	M	13	0	Home		58	M	Intracerebral hemorrhage	2	CE + TCD
3		62	M		47	M	5	19	Died		40	F	Cardiac arrest	5	CE
4		41	M		29	M	7	9	Home		76	M	Cardiac arrest	8	CE
5		33	F		88	F	4	9	Died		20	M	TBI	6	CE
6		33	M		33	M	14	7	Home		34	M	Intracerebral hemorrhage	8	CE
7		35	M		39	M	3	4	Died		52	F	Intracerebral hemorrhage	23	CE
8		38	M		52	F	8	18	Died		47	M	TBI	11	CE
9		27	M		50	M	12	0	Home		65	M	Cardiac arrest	7	CE
10					86	F	5	9	Died		48	M	Intoxication	4	CE
11					67	M	11	10	Died		46	M	Intoxication, cardiac arrest	4	CE
12					54	F	12	3	Home		61	M	Intoxication, cardiac arrest	3	CE + CT perfusion
13					63	M	13	0	Home		58	F	Intracerebral hemorrhage	20	CE
14					24	M	7	0	Home						
15					46	M	5	0	Home						

No. - patient number, M - male, F - female, GCS - Glasgow Coma Scale score on admission, MLS – midline shift assessed from CT scan, TBI - traumatic brain injury, CE - clinical evaluation, CT - computed tomography, TCD- transcranial Doppler.

Cerebral edema group was characterized in details previously^34^.

**Figure 1:**
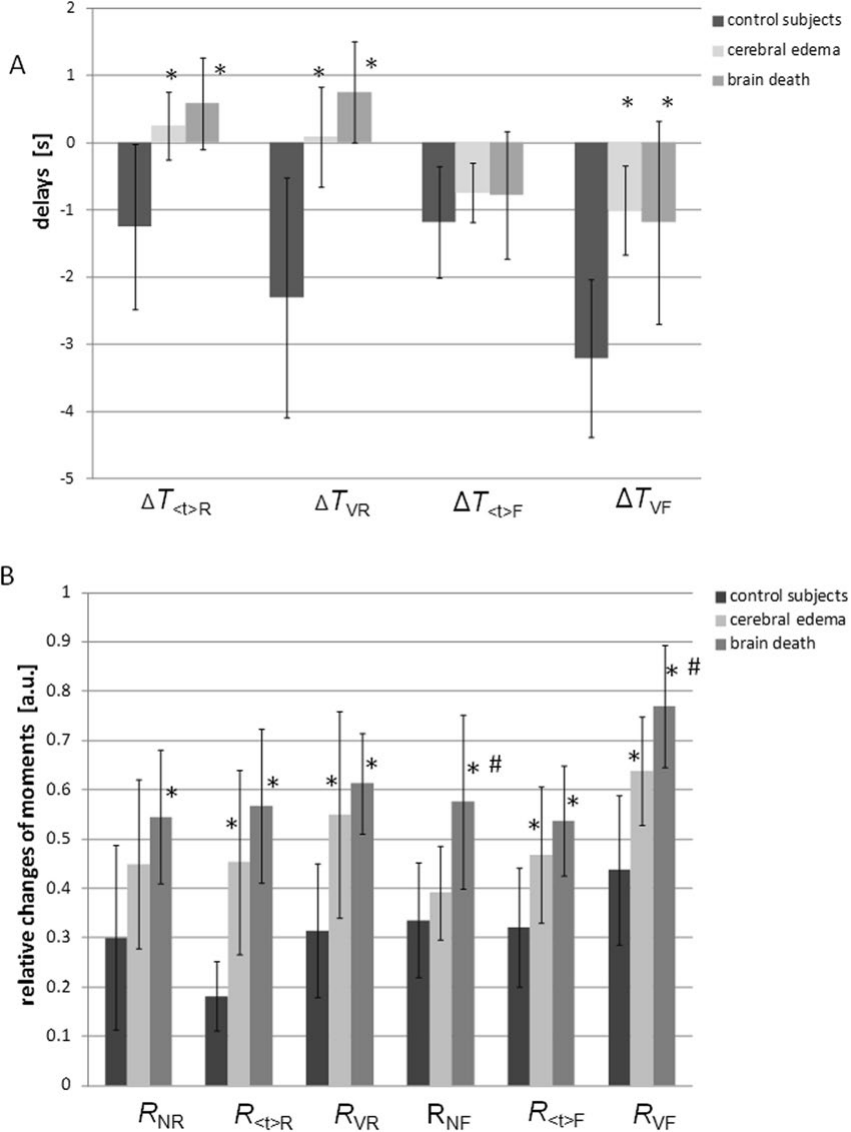
Mean values and standard deviations of perfusion variables obtained in control subjects, patients with cerebral edema, and patients with brain death. (**A**) ICG inflow-related variables (Δ*T*) and (**B**) ICG outflow-related variables (*R*). *Differences between the cerebral edema or brain-dead group and the control group (p < 0.05). ^#^Differences between the cerebral edema and brain-dead groups (p < 0.05).

The results of the measurements carried out in patients with brain death were analyzed together with the results obtained in the control group (healthy volunteers) and those obtained from patients with posttraumatic cerebral edema (the measurements for these patients and the control group were performed during our previous study^34^; Table 1). Univariate comparison of all variables related to inflow of the dye (Δ*T*_<t>R_, Δ*T*_VR_, Δ*T*_<t>F_, and Δ*T*_VF_) and its outflow (*R*_NR_, *R*_<t>R_, *R*_VR_, *R*_NF_, *R*_<t>F_, and *R*_VF_) between the groups of subjects is presented in Fig. 1. Most differences were found between the disease groups, namely the cerebral edema and brain-dead groups, and the control group. Only *R*_NF_ was different between the brain-dead and cerebral edema groups, while *R*_VF_ was different in all three groups.

**Figure 2:**
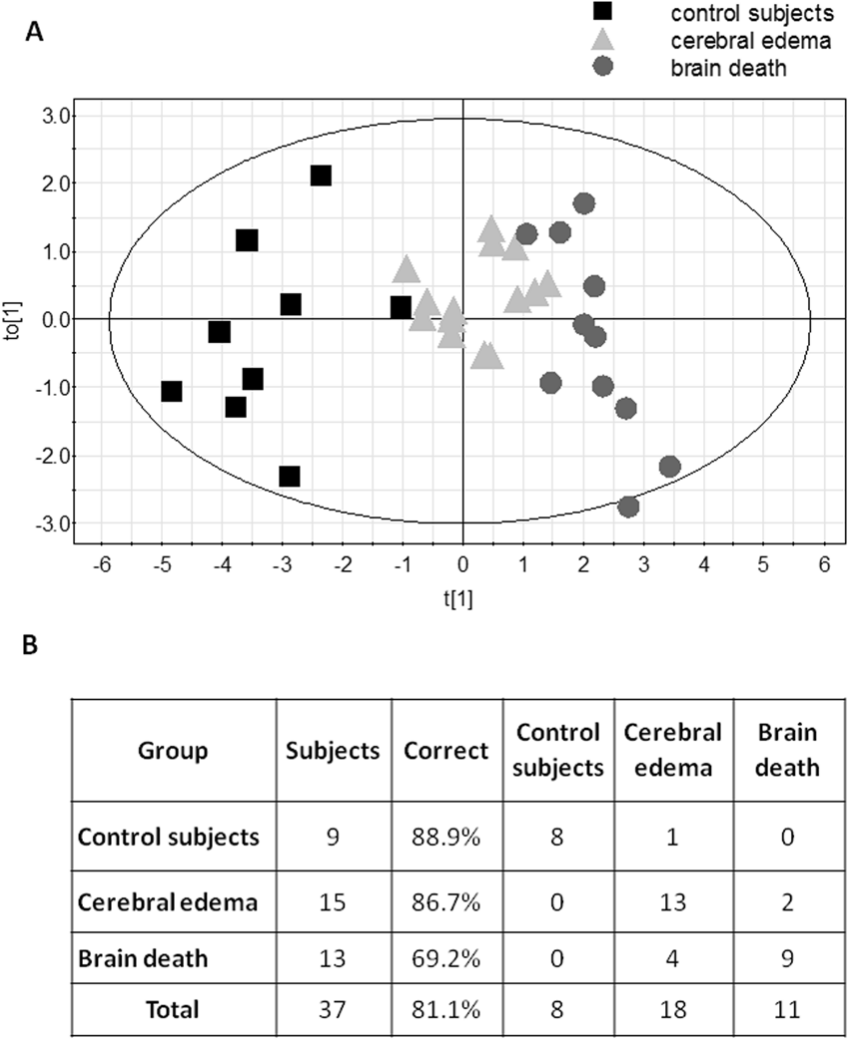
(**A**) Orthogonal partial least squares-discriminant analysis (OPLS-DA) scores plot of ICG inflow and outflow variables showing separation between the three groups. The control subjects are on the left side of scores plot, patients with posttraumatic cerebral edema are in the middle, and patients with brain death are on the right side of the scores plot (model 1). The score vector t_o_[1] represents within-group variation in the orthogonal component, while the score vector t[1] represents between-group variation in the predictive component. The group discrimination is seen along the t[1] axis. The ellipse represents Hotelling T^2^ with 95% confidence in the scores plots. (**B**) Misclassification table of subjects classified to different study groups using model 1.

We used MVA for further analysis. The OPLS-DA classification model was first constructed using data from all subjects: healthy volunteers (n = 9), patients with posttraumatic edema (n = 15), and patients with brain death (n = 13) (model 1). We included all ICG inflow (*ΔT*) and ICG outflow (*R*) variables. Mean centering and autoscaling were performed before analysis. Data from one patient with cerebral edema and two patients with brain death were removed as strong outliers from the model. This procedure allowed us to build a model consisting of one predictive and one orthogonal component (Fig. 2A). The scores plots obtained from the perfusion variables indicate significant separations between groups of subjects along the predictive component (horizontal axis). R^2^ was 0.67 (good data fit) and Q^2^ was 0.25 (moderate data prediction). Overall, 30 of 37 subjects (81.1%) were classified correctly (8 out of 9 control subjects, 88.9%; 13 out of 15 patients with edema, 86.7%; and 9 out of 13 patients with brain death, 69.2%; p < 0.0001; Fig. 2B). The validation test (CV-ANOVA) confirmed that the model was valid (p = 0.024). The sensitivity, specificity, accuracy, and the significantly distinguishing perfusion variables (VIP > 1) for model 1 and the following models are presented in Table 2.

**Table 2:** Sensitivity, specificity, accuracy, and most influential variables (VIP > 1) of the four analyzed OPLS-DA models.

	Group comparison	Sensitivity^a^	Specificity^b^	Accuracy^c^	Most influential variables (VIP value)
Model 1	Control vs. cerebral edema	80%	92.9%	87.5%	*R*_VF_ (1.21) Δ*T*_VF_ (1.15) *R*_<t>R_ (1.14) Δ*T*_VR_ (1.13) Δ*T*_<t>R_ (1.05) *R*_VR_ (1.04)
	Control vs. brain dead	66.7%	90%	77.3%	
	Cerebral edema vs. brain dead	76.5%	81.8%	78.6%	
Model 2	Cerebral edema vs. brain dead	92.9%	85.7%	89.3%	*R*_NF_ (1.53) *R*_VF_ (1.46) Δ*T*_VR_ (1.26) *R*_<t>R_ (1.16)
Model 3	Cerebral edema vs. brain dead	86.7%	84.6%	85.7%	*R*_NF_ (1.24) *R*_VF_ (1.2) *R*_<t>R_ (1.19)
Model 4	Cerebral edema vs. brain dead	77.8%	90%	82.1%	*R*_NF_ (1.54) *R*_VF_ (1.43)

^a^Proportion of those with the target condition (brain death, or cerebral edema in case of cerebral edema vs. control) who are correctly classified (true positives) using the optical method measurements.

^b^Proportion of those without the target condition who are correctly classified (true negatives) using the optical method measurements.

^c^Accuracy is the proportion of true results (both true positives and true negatives) of the total number of cases examined.

**Figure 3:**
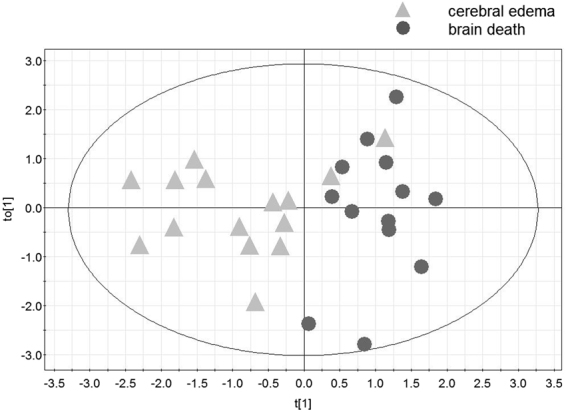
Orthogonal partial least squares-discriminant analysis (OPLS-DA) scores plot of ICG inflow and outflow variables showing separation between the two groups: patients with posttraumatic cerebral edema and those with brain death (model 2). t_o_[1] represents within-class variation in the first orthogonal component, while t[1] represents between-class variation in the first predictive component. The ellipse represents Hotelling T^2^ with 95% confidence in the scores plots.

We constructed consecutive OPLS-DA classification models using data from the two patient groups (cerebral edema and brain-dead, as presented previously)^35^ because their differentiation represents a more real clinical dilemma. Using 10 variables (as above) and OPLS-DA analysis, we were able to construct a model consisting of one predictive and two orthogonal components (Fig. 3) (model 2). For this analysis, R^2^ was 0.56 (good data fit) and Q^2^ was 0.52 (good outcome prediction). Overall, 89.3% of patients were correctly classified (13 out of 15 patients from the cerebral edema group, 86.7%; and 12 out of 13 patients from the brain-dead group, 92.3%; p < 0.00001). CV-ANOVA was applied to confirm that the model was valid (p = 0.013).

We tested whether we can yield OPLS-DA models only for separate inflow and outflow variables. The model built using inflow variables was not valid (p = 0.39). The model based on outflow variables consisted of one predictive component and had an R^2^ of 0.47 and a Q^2^ of 0.26 (model 3). Data obtained from one patient was removed from the model as a strong outlier. The model was valid (p = 0.028). Overall, 85.7% of the patients were correctly classified (13 out of 15 patients with cerebral edema, 86.7%; and 11 out of 13 patients with brain death, 84.6%; p < 0.001).

Finally, we compared optical measurements based on diffuse reflectance to those based on fluorescence. The model built with reflectance variables was not valid (p = 0.22). The model built with fluorescence variables consisting of one predictive component was valid (p = 0.046) (model 4). In this model, R^2^ was 0.34 and Q^2^ was 0.26. Overall, 82.1% of the patients were classified correctly (14 out of 15 in the edema group, 93.3%; and 9 out of 13 in the brain-dead group, 69.2%; p = 0.008).

## Discussion

In this study, we demonstrated that an optical method based on the tracking of an optical dye (ICG) bolus through the brain was able to discriminate between healthy subjects, patients with posttraumatic edema, and patients with brain death, with moderate accuracy. The study was designed to assess the robustness of the method for distinguishing different stages of cerebral perfusion insufficiency, from normal (in controls) to moderate (in patients with cerebral edema) to complete cessation of flow. However, here we made the assumption that the CBF disturbances observed are in fact reflective of those observed in patients with certain diagnosis. This assumption should hold true in healthy controls where perfusion abnormalities are not expected. In contrast, cerebral edema can result in a wide range of cerebral perfusion insufficiencies ranging in severity from mild to severe, depending on the progression of pathology. In this study, some subjects with cerebral edema were classified into the brain-dead group (Figs 2 and 3). We cannot exclude the possibility that CBF was highly compromised in these patients in the volumes of tissue wherein the measurements were carried out. In addition, some patients with brain death were classified into the cerebral edema group. Previous studies have shown that, depending on the method used for the assessment of cerebral perfusion, CBF remains to some extent preserved in certain patients with cerebral death^36–38^. This effect was seen in 20% of cases in the cortical branches of the middle cerebral artery and in 41% of cases in the anterior cerebral artery^39^. Four and one patient out of the 13 patients with brain death were classified into the cerebral edema group based on models 1 and 2, respectively (Figs 2 and 3). This pathophysiological variation may in part explain the overlap between the bordering groups in our classification scatter plots. However, this reasoning remains speculative, as the results obtained with the optical method were not compared to those obtained with any other cerebral perfusion method. Notwithstanding these drawbacks, our OPLS-DA model 1 (comparing the three groups) had good data fit and moderate data prediction, while model 2 (comparing the cerebral edema and brain-dead groups), which addressed the more common clinical challenge, had both good data fit and good prediction.

Univariate analysis showed that only the *R*_VF_ variable was able to distinguish all study groups. In further analysis, we checked whether the combination of variables would increase separation of the groups in the multivariate analysis. OPLS-DA is a technique that successfully copes with large numbers of predictors (here, ten perfusion variables) and few observations. This method also reveals the most influential variables that account for group separation. In this study, the variables common to models 1 and 2 that played the most important roles (VIP > 1) in differentiating between the groups were *R*_VF_, *R*_<t>R_, and Δ*T*_VR_. However, slight differences in the influences of other variables on the considered models were observed. Because most influential variables described both ICG inflow and outflow phenomena, as well as depended on diffuse reflectance and fluorescence, we performed additional data analysis to determine the usefulness of different groups of variables measured via TR-NIRS.

In our study, it was not possible to directly calculate CBF because arterial input function (AIF) from artery supplying brain was not available as it is in classical perfusion measurements^40^. However, we developed other indices of perfusion that are easier to estimate and rely only on TR-NIRS signals^34^. The variables that depend on ICG inflow were determined based on the results of previous physiological experiments wherein it was shown that the inflow of the tracer to the brain precedes inflow to the extracerebral tissues^26,41^. Further, decreased cerebral perfusion resulted in delayed inflow of the tracer to the brain^42,43^. Thus, temporal differences in inflow to the extracerebral and intracerebral tissues can reveal brain perfusion insufficiencies. This concept was tested with perfusion computed tomography measurements, where inflow of tracer in the extracranial artery could successfully serve as AIF^44^. Based on these theoretical considerations and the results of previous experiments, we assumed that the signals of the number of photons *N* are sensitive mostly to the inflow of ICG to the superficial tissues and that the higher-order statistical moments signals <*t*> and *V* are mostly sensitive to ICG inflow to cerebral cortex^26,45,46^. Thus, the signals due to changes in the number of photons *N* can be considered as surrogates of AIF, and the temporal delay between the appearance of the bolus as a change in the signals from the number of photons *N* and the higher-order statistical moments contain information on cortex perfusion.

We were not able to use OPLS-DA to create a valid model with only inflow-related variables. This may be associated with the strong heterogeneity of the blood flow in extracerebral tissues. However, it was possible to build a valid model using only outflow variables (model 3). Several studies in patients with TBI have shown that compromised microcirculation is associated with prolonged tracer outflow from the brain, as determined in CT studies^42,43^. Thus, our outflow variables may reflect microcirculation insufficiency provided that the blood-brain barrier remains intact. Furthermore, these variables would lead to an even higher washout ratio *R* of ICG when there is cessation of cerebral circulation, as the signal in this case would originate solely from extracerebral tissue, where relatively high extravasation (and prolonged ICG washout) is a physiologic phenomenon.

Comparison between diffuse reflectance and fluorescence measurements (model 4) revealed that the analysis of signals related to fluorescence excited in ICG appear to be superior. These results are in line with those of our previous studies wherein we analyzed the advantages of fluorescence signal monitoring with respect to diffuse reflectance^47,48^. To sum up our results, we found that washout-related variables, variables computed from fluorescence signals, and those depending on higher order statistical moments of the DTOF or DTA (variance) are most meaningful. However, as expected, in our statistical model, the maximum class separation was achieved when we used all variables and the reduction of any variable resulted in decreased predictive value of R^2^.

An ancillary test confirming the cessation of CBF must have relatively high sensitivity and nearly 100% specificity (i.e., no “false positives”) because patients with preserved cerebral circulation cannot be classified as dead by the test under consideration. Optical methods tested in this study did not achieve these goals, as they had sensitivities of 67–93% and specificities of 82–86%. However, these parameters would place the tests used here alongside other currently investigated methods for brain death confirmation, i.e., computed tomography angiography (sensitivity, 63–87.5%^37,39,49^; or diffusion-weighted magnetic resonance imaging (sensitivity 90%)^50^. Other methods that have higher sensitivity and specificity have limited accessibility (Xenon CT)^1^, are difficult to perform (magnetic resonance angiography)^51,52^ or are operator-dependent (transcranial Doppler; sensitivity 88%, specificity 98%)^53^. We have shown that optical methods based on TR-NIRS are feasible during an ICU stay, available at the bedside, and have promising preliminary results. Thus, this technique, as used here, opens a new direction in the search for an ancillary test based on the evaluation of cerebral perfusion.

As mentioned earlier, a serious limitation of this study was that we compared measurements of perfusion disturbance to clinical evaluation only and not to any other perfusion assessment method, as no reference perfusion method was available during the study. Furthermore, even though we achieved satisfactory data fit to our model, the number of patients was relatively low and the results changed considerably when the controls were removed from the model. However, brain death declaration is not a frequent procedure and experiments regarding this legal procedure face extraordinary challenges related to the bereavement of relatives and eventual preparation for organ donation. The low number of patients in each group did not allow us to adjust for age and sex. Previous studies have demonstrated that CBF appears to decrease slightly with age and is higher in women than in men^54,55^. A few studies showed that such differences in cerebral cortex perfusion were even more pronounced^56^. Another factor unavailable during our measurements was the patient’s cardiac output, which influences CBF^57^ and could differ among patients and study groups, even though all the participants were hemodynamically stable.

Additionally, there are some general limitations of optical methods that also apply to confirmation of brain death^58^. The most important of these limitations is the limited depth of light penetration, which leads to a limited volume of the brain being interrogated by the photons. Deeper penetration of light has been achieved by increasing source-detector separation in experimental settings only^59^. Inaccessibility of deeper brain structures to optical methods as an absolute obstacle in brain death determination may be debatable. A recent international survey has demonstrated that the most common ancillary tests currently used is the EEG^60^, which in fact is based on assessing only cortical neuronal activity^13^. Furthermore, some studies have reported a close relationship between EEG activity and NIRS measurements^61^. However, the traditional view that ancillary tests based on perfusion must detect cessation of CBF in the whole brain^17^ would lead to the use of optical methods as simply screening tools rather than real confirmatory tests. Ideally, optical methods should be able to distinguish extra- and intracerebral perfusion disturbances separately to confirm the cessation of intracranial blood flow, and also detect preserved extracranial perfusion. Our method could, at best, compare temporal delays between inflow to extra- and intracerebral tissues, but not direct perfusion values, in these layers.

Limited spatial resolution of the optical methods can be overcome to some extent by application of multiple optodes^62^. Such distribution of optodes might lead to some measurement challenges related to uneven skull translucency, as recently described^63^. However, our experiments showed that the optode placement in critically ill patients does not need to be restricted to the forehead, as typical in currently available commercial devices.

Apart from the technological limitations, other constrains related to patient condition may appear while using optical methods. The measurements are not possible over cerebral cortex covered by extracerebral hematoma, which exhibits high light absorption properties^64^. Other difficulties are present in patients with liver insufficiency, as it strongly affects ICG kinetics^65^, especially in the washout phase. Cerebral perfusion algorithms that take into account measuring peripheral ICG elimination by means of transcutaneous and non-invasive pulse dye densitometry might be useful in patients with liver insufficiency^24,66^.

In conclusion, this first study assessing an optical method in combination with an intravascular contrast agent for confirmation of brain death proved that the method is feasible in the demanding ICU environment and had promising initial results. However, at its current stage of development, the accuracy of the method did not reach the high standards required for brain death confirmation. Critical issues that need to be resolved are the limited depth of light penetration, the lack of confirmation of accuracy against other methods evaluating cerebral flow cessation, and the absence of absolute parameters for cerebral perfusion.

## Methods

This case-control feasibility and diagnostic accuracy study is a continuation of our previous experiments^34^ wherein we used TR-NIRS and an optical contrast agent to assess CBF disturbances in patients with traumatic brain injury. In the present study, we included patients with the clinical declaration of brain death. The study was approved by the Ethics Committee of the Medical University of Warsaw, Poland (KB-O/6/10), and was conducted in accordance with the principles laid out in the Declaration of Helsinki and national regulations. We obtained verbal consent for the experiment from the relatives of all patients after explicit explanation of the aims and methods of the study. The study was conducted in the ICU of a secondary level of care hospital in Warsaw, Poland. The hospital has a regional referral center for toxicology. We prospectively included all adult patients (≥18 years of age) in whom brain death was legally declared after clinical evaluation who had stayed in the ICU between September 2009 and January 2012. Clinical evaluation was performed according to national guidelines and in agreement with the international standard for determining brain death^67,68^. During the study period, national regulations stated that in order to determine brain death, the following conditions needed to be met: a suitable observation period before clinical examination (>6 hours and >12 hours for primary and secondary brain injury, respectively), brain injury of established etiology, irreversible coma, and requirement of mechanical ventilation. Furthermore, the exclusion criteria for declaring brain death were: influence of drugs, intoxication or narcotic agents, hypothermia, and metabolic and endocrine disturbances. Only under such circumstances could clinical examination for determining brain death, including brainstem reflexes and apnea testing, be performed and repeated after 24 hours (if no ancillary test was additionally performed)^68^. Intoxicated patients were first admitted to the toxicology center, underwent detoxification (e.g., hemodialysis), and were transferred to the ICU when the inclusion criteria for brain death determination were met and none of the exclusion criteria were applicable.

Patients who were hemodynamically unstable, had liver insufficiency (bilirubin level >51.3 µmol/L [3 mg/dL], international normalized ratio (INR) > 1.4)^65^, and those without informed consent from relatives were excluded from the study. All consecutive patients with the suspicion of brain death were evaluated and screened for study eligibility by the first author (W.W.). The number of patients available during the study period determined the sample size. In order to assess the results from patients with brain death, we compared them to those obtained from healthy volunteers and patients with posttraumatic cerebral edema. The ICG-tracking measurements in these patients have been reported in our previous study^34^. In each case, the written informed consent was obtained from the volunteer, patient or the patient’s legally authorized representative. Posttraumatic cerebral edema was identified using computed tomography (CT) not more than 24 hours before the optical measurements. It was represented on imaging as the loss of sulci, compression of the basilar cisterns, and flattening of the ventricular margins. We have reported this study following the Standards for Reporting of Diagnostic Accuracy (STARD) guidelines^69^ where applicable.

### Instrumentation

Measurements of the inflow and washout of the optical contrast agent passing through the brain were carried out using a prototype TR-NIRS instrument built in-house and described in detail elsewhere^29,70^. The system was equipped with two laser diodes operating at λ = 760 nm and two identical optode holders, each consisting of 2 source fibers and 2 detection bifurcated fiber bundles. Thus, each optode-holder consisted of 4 source-detector pairs, allowing for simultaneous measurements of both diffuse reflectance and fluorescence optical signals. The distance between each source and detector was 3 cm. TR-NIRS allows us to record the distributions of times of flight (DTOFs) of diffusely reflected photons and the distributions of times of arrival (DTAs) of fluorescence photons. These distributions were acquired using Time Correlated Single Photon Counting electronics combined with photomultiplier tubes and filters allowing us to select signals of diffuse reflectance and fluorescence. The DTOFs and DTAs were acquired at a sampling frequency of 10 Hz. The measurements were carried out when the single photon count rate was not lower than 10^6^ photons per second for a single source-detector pair in the diffuse reflectance channels.

### *In vivo* measurements

Measurements were performed after brain death declaration when the patients were still mechanically ventilated and before transplantation procedures, if they were planned. Two hand-held optode holders were placed bilaterally on the surface of the head in such a way that the center of the square formed by the tips of source fibers and the detecting fiber bundles were positioned at the C3 and C4 locations according to the 10–20 electroencephalography (EEG) system. In patients who underwent craniotomy, measurements were performed just over the contralateral hemisphere. Hair under the optodes was manually moved away. The 5-mg bolus of ICG (Pulsion, Germany) was dissolved in 5 mL of water and rapidly injected intravenously. This was immediately followed by a 10-mL normal saline flush. Data acquisition began 30 seconds prior to injection and continued for 5 minutes.

### Data analysis

The 0^th^, 1^st^, and 2^nd^ statistical moments of the DTOFs and DTAs, i.e. the total number of photons *N*, mean time of flight (arrival) <*t*>, and variance *V*, respectively, were calculated in the Matlab environment as described previously^71^. The time courses of the statistical moments were smoothed using the moving average method with a window width of 3 seconds. Averaged signals of the statistical moments were calculated from signals obtained in 4 channels (one optode holder) located on the affected (after trauma) hemisphere for patients with edema. We obtained averaged signals from 8 channels (two optode holders) located on both hemispheres in patients with brain death and control subjects, in whom we did not expect differences between the hemispheres.

We have developed several measures of ICG kinetics in the brain. To quantify ICG inflow, we used an algorithm based on the analysis of temporal delays (Δ*T*) between the maxima of the derivatives of the changes in statistical moments (Δ<*t*> and Δ*V*, which reflect the inflow of the contrast agent into the cerebral tissue) in relation to the maximum of the derivative of the changes in statistical moments of Δ*N* (which reflects inflow of the contrast agent into the extracerebral tissue). The same method was used in our previous study^34^. The concept of temporal delays between the maximum of the derivative of the 0^th^ order of statistical moment (*N*) and the higher order of statistical moments (<*t*> and *V*) is based on the differences in sensitivity of the consecutive moments to changes in absorption appearing at different depths^26^. These temporal delays describe the kinetics of inflow of the dye to the brain and represent variables related to cerebral perfusion: Δ*T*_<t>R_ and Δ*T*_VR_ for measurements of diffuse reflectance, and Δ*T*_<t>F_ and Δ*T*_VF_ for fluorescence measurements.

**Figure 4:**
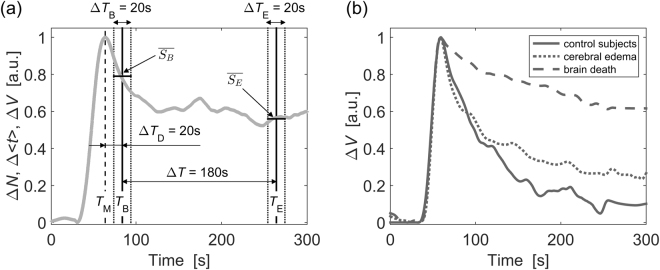
(**a**) Algorithm for the data analysis used in the assessment of the ICG washout ratio. *T*_M_ - time of maximum amplitude of the signal, *T*_B_ – time at which the baseline signal level was obtained (20 seconds after *T*_M_). Averaged signal amplitude $\overline{{{\boldsymbol{S}}}_{{\boldsymbol{B}}}}$ was calculated within the Δ*T*_B_ period. *T*_E_ – time at which the late signal level was obtained (180 seconds after *T*_B_). $\overline{{{\boldsymbol{S}}}_{{\boldsymbol{E}}}}$ - averaged signal amplitude within the Δ*T*_E_ period. (**b**) Examples of normalized time courses of variance of the statistical moments of the distributions of times of arrival (DTAs) of fluorescence photons (ΔVF) acquired during injection of ICG in a control subject, patient with brain edema and patient with brain death. *R*_VF_ variables calculated from these signals (ΔVF) were most significant in study groups differentiation (please, see *Results* section). For presentation purposes signals obtained in different subjects/patients were aligned by their maximum value.

Assessment of the ICG washout rate was conducted using an algorithm based on the analysis of amplitude changes in the statistical moments related to the clearance of the dye (see Fig. 4)^72^. We first normalized the time courses of all statistical moments in such a way that the minimum value was subtracted and the resulting signal was normalized by its maximum value. To assess relative changes in amplitudes of the signals of statistical moments in the washout phase, we averaged the amplitudes of the signals $\overline{{S}_{B}}$ (occurring at Δ*T*_D_ = 20 seconds after the signal reaches its maximum) and $\overline{{S}_{E}}$ (occurring 180 seconds later). The signals of the moments were averaged for two 20-second time windows (Δ*T*_B_ and Δ*T*_E_). The relative changes *R* in the statistical moments were calculated according to the following formula:

1 $$R=\frac{\overline{{S}_{B}}-\overline{{S}_{E}}}{\overline{{S}_{B}}}$$

where $\overline{{S}_{B}}$ represented the averaged amplitude of the change in the signal calculated for the time period Δ*T*_B_ and $\overline{{S}_{E}}$ was the averaged amplitude of the change in the signal calculated for the time period Δ*T*_E_. This algorithm was used to analyze the signals of Δ*N*, Δ <*t*>, and Δ*V* for the DTOFs and DTAs. This was in turn used to calculate relative changes in *R*_NR_, *R*_<t>R_, and *R*_VR_ for diffuse reflectance measurements and *R*_NF_, *R*_<t>F_, *R*_VF_ for fluorescence measurements.

### Statistics

First, we performed univariate data analysis for all measured optical perfusion variables using a one-way analysis of variance (ANOVA) followed by post-hoc Dunn’s or Holm-Sidak analysis. We performed multivariate analysis (MVA) to explore the data further. We used orthogonal partial least squares-discriminant analysis (OPLS-DA), which is an MVA projection method. OPLS-DA is a classification method based on the PLS regression algorithm^73^. This technique aims to maximize the covariance between the independent variables, which is referred to as X (matrix of original data; here, optical parameters related to cerebral perfusion), and that for the dependent variable, which is referred to as Y (matrix of responses; here, a corresponding clinical condition – brain death, cerebral edema, or control group). The method dissects the systematic X variation into two parts, one that is correlated to Y (called the predictive component, reflecting the between-group variation), and another that is orthogonal (uncorrelated) to Y (called the orthogonal component, reflecting the within-group variation). This method, which allows us to build a classification model, also optimizes separation between groups of subjects and provides a visual interpretation of such separation through an easily interpretable scores plot^74^. The ellipse drawn on the scores plot represents the Hotelling T-square test with 95% confidence in the model. Scores located significantly outside the Hotelling T-square ellipse are removed as outliers. Scores inside the Hotelling T-square ellipse representing observations on the plot that are close to each other are more similar than observations distant from each other. The separation of groups of observations is seen on the plot if one group of scores can be clustered and distinguished from another clustered group of scores in the predictive component (horizontal axis of the plot). Distance between scores in the vertical direction is indicative of within-group variation. Although OPLS-DA is used mostly for spectroscopic data analysis, e.g., metabolomics, it has also been recently used in several neuroimaging studies^75^. This method allowed us to construct a classification model to distinguish between control subjects, patients with cerebral edema, and patients with brain death. The model performance was described by R^2^, which explains the total variation in the data and estimates goodness of fit, and Q^2^, which is an internal cross-validation parameter estimating the goodness of the prediction. We adopted the limit of good predictive capability for the OPLS-DA model, as described elsewhere^76^. The model was positively validated when R^2^ and Q^2^ were equal or greater than 0.5.

To identify the most important of the 10 available cerebral perfusion variables (4 variables describing the inflow phase [Δ*T*] and 6 variables describing the washout phase [*R*]), we calculated Variable Importance in the Projection (VIP) values. Variables with VIP values higher than 1 were considered significant for discrimination between groups, and higher VIP values indicated greater contribution of the variable to the discrimination between groups. We checked the reliability of the model using ANOVA of Cross-Validated predictive residuals (CV-ANOVA) and the jackknifing method. The Fishers test was used to assess significance of the group separation and the validity of the method. P values < 0.05 were considered significant. Univariate analysis was performed using Statistica v.10 (StatSoft, Inc.) and MVA was performed using SIMCA-P v.12 software (Umetrics AB, Sweden).

### Data availability

The datasets generated and analyzed during the current study are available from the corresponding author on reasonable request.
